# A review of health and wellness studies involving Inuit of Manitoba and Nunavut

**DOI:** 10.1080/22423982.2020.1779524

**Published:** 2020-06-16

**Authors:** Ashley Hayward, Jaime Cidro, Rachel Dutton, Kara Passey

**Affiliations:** a Student Peace and Conflict Studies, University of Winnipeg, Winnipeg, Canada; b Anthropology, University of Winnipeg, Canada Research Chair, Health and Culture, Winnipeg, Canada; c Manitoba Inuit Association, Winnipeg, Canada; d Development Practice: Indigenous Development (MDP) Student, University of Winnipeg, Winnipeg, Canada

**Keywords:** Inuit, health and wellness, Manitoba, chronic conditions and diseases, population and preventative health, the determinants of Inuit health, Nunavut, environmental health, food security and nutrition-based health, community directed research

## Abstract

The purpose of this review is to summarise past Inuit health and wellness studies in Manitoba and the Kivalliq region of Nunavut to provide a snapshot of the types of studies available and identify the gaps in knowledge. Research to date has largely been disease-based and often provides comparisons between Indigenous and non-Indigenous people. Distinct Inuit experiences are rarely written about from an Inuit perspective. However, Inuit Tapiriit Kanatami, the national organisation of Inuit in Canada, and Pauktuutit Inuit Women of Canada have been leaders in strengths-based community research and publications that address priorities determined by the Inuit, including the 2018 Inuit Tapiriit Kanatami document *National Inuit Strategy on Research* (132).

## Introduction

The Inuit, an Indigenous group of Canada, comprise about 65,000 of the national population [1]. There are four Inuit regions in Canada, and the majority of Inuit live in fifty-three communities in a geographical location that is often referred to by the Canadian Inuktitut term *Inuit Nunangat*. This term for “homelands” includes land, water, and ice [2]. All Inuit populations despite their country of origin have “significant gaps between their health status and those of broader national populations” [3 p.1]. Despite the recognition of health disparities [4–13], “circumpolar [Arctic] regions [including parts of the USA (Alaska), Russia, and Denmark (Greenland)] are overlooked by the World Health Organization” [4 p.1248]. Although the health challenges faced by Inuit can be similar across their homelands in the circumpolar region, the types of interventions and health care systems vary among each country. So, although Inuit in each of these regions share commonalities through cultural knowledge and language, they remain distinct groups.

Health and wellness are broad concepts that require more rigorous definition and nuance in the mainstream research as they pertain to Indigenous populations. A more recent shift in health literature has moved towards the social determinants of health (income and social status; social support networks; education; employment/working conditions; social environments; physical environments; personal health practices and coping skills; healthy child development; gender; and culture) as a way of examining the impact of external socioeconomic forces on the health of individuals and communities [14–19]. For Indigenous peoples, health is much broader than the absence of disease; it is our overall well-being, that determines our physical health in the future. As health historian Daschuk states, “Health as a measure of human experience cannot be considered in isolation from the social and economic forces that shape it” [20 p.ix]. This holistic approach has been recognised and practiced by Indigenous people for generations, but has faced numerous challenges from the forces of colonisation. From 2007 to 2010, the Qikiqtani Truth Commission interviewed Inuit in Canada and produced the *Qikiqtani Truth Commission: Community Histories 1950–1975* and *Qikiqtani Truth Commission: Thematic Reports and Special Studies*. These reports document the Inuit experience and Canadian government decisions during the 1950 s, 60 s, and 70 s as told by Inuit. These governmental policies, which included mandatory formal education for children with a curriculum that had no relevance to life in the North, impacted the lives of Inuit specifically in the Qikiqtani region of Nunavut, and are still felt today [21]. Hunting regulations established by the government enforced laws based on southern conservation priorities that strictly defined the hunting seasons with restricted numbers of animals rather than Inuit knowledge, traditional law, customs, and practices [21].

Colonisation has impacted, and continues to impact, the Inuit in Canada, including the many Inuit who were moved from family-based land camps to permanent crowded settlements [22]. Inuit also experienced residential and day schools [22], a central component in the federal government’s “Aboriginal Policy” and goal of assimilation [23]. Residential or day schools were government-sponsored, church-run schools designed to “remove” or “kill the Indian out of the child” [24], and have been labelled as “cultural genocide” by the Truth and Reconciliation Commission of Canada: “Cultural genocide is the destruction of those structures and practices that allow the group to continue as a group” [23 p.1]. Education is a social determinant of health, and the residential school system continues to have an effect on Inuit health today. Nineteen per cent of Inuit had a college diploma in 2016, which is an increase from previously reported statistics in 2006 [1], but is still lower than the national average of 22.4% of the Canadian population aged 25 to 64 [25]. The location of postsecondary institutions, which are usually in major cities and urban areas, may also affect the rates of postsecondary educational attainment [26]. Educational attainment levels, and in particular high school competition rates, are lowest among Inuit communities and First Nations people living on reserves, which correlates with the communities with the highest percentages of descendants of residential school survivors [23]. Furthermore, educational attainment levels go on to shape employment opportunities and income levels [26].

Overall, the Inuit are the youngest of the three Indigenous groups in Canada (the other two being First Nations and Métis), with an average age of 27.7 years [1]. The young population is at risk for poor mental health with 101 per 100,000 Inuit youth aged 10 to 19 living in Inuit Nunangat experiencing acute-care hospitalisations for intentional self-harm [1]. These rates are associated with colonialism and imperialism [27]. The isolated or remote geographical location also impacts health and wellness in a variety of ways including recruitment and employment opportunities for trained medical professionals and language barriers when seeking care [28,29]. Many remote communities are “economically disadvantaged and isolated” [28 p.192] and experience “unequal access to and lower utilization of health care services” [28 p.196]. Although there is no universal rule for what defines a “remote” location, in this context, a remote geographical region is defined as having a relatively low population density (although sometimes they do have some high concentrations of population density in small pockets of the region) [30].

Despite the resiliency of the Inuit population, including the maintenance of their language^1^ ^1^Language is an important component of culture, which is included in the framework of social determinants of health [6], and Inuit living in Inuit Nunangat are more likely to speak an Inuit language, such as Inuktitut, than those living outside the region. and commercial hunting practices that have “a significant impact on Nunavut’s GDP, add key infrastructure, and foster self-reliance and employment for Inuit people” [31], it is important to recognise the unique challenges of the geographical location, including access to healthcare and food security. According to McDonald and Trenholm [32], only 31% of Inuit live within 50 kilometres of a regional hospital that has limited specialised health services. Between 2008 and 2017, the province of Manitoba retained 57.4% of its trained physicians, with merely 14.9% practicing in rural or remote communities [33]. This creates Northern and remote communities that are medically underserviced with limited access to primary care providers [34]. Furthermore, because many communities are remote, the costs of goods and services are high, placing economic stressors on residents [35]. Among Inuit living in Inuit Nunangat, 52% of adults experienced food insecurity defined as “not having enough food to eat due to lack of money.” [21 p.1]. Food insecurity is the highest in Nunavut where 70% of households are food insecure [36]. Experiences living in poverty also influence food selection, and in 2017, the unemployment rate in Nunavut was 12.6% [37]. The cost of harvesting supplies and the decline in some animal species, such as caribou, are also examples of factors contributing to food insecurity [36].

The purpose of this paper is to provide a comprehensive scoping review of health and wellness literature related to Inuit in the province of Manitoba and the Kivalliq region, one of the three main regions within Nunavut. The primary aim of this work is to support the development of a community-driven research project to support Indigenous self-determination in research. The Manitoba Inuit Association (MIA) is one of the partners in the Canadian Institutes of Health Research-funded Network Environment for Indigenous Health Research (NEIHR), and this scoping study will support the organisation in identifying gaps in knowledge in health and wellness for Inuit in Manitoba and the Kivalliq region. MIA provides programmes and services to Inuit living in or visiting Manitoba while promoting Inuit values, community and culture. MIA established the research questions based on parameters relevant to their organisation and the population their mission supports. The research questions, designed to include the greatest breadth of scholarship, are: 1) what type of health and wellness research has been conducted on/with the Inuit in the identified regions?; 2) what type of health and wellness resiliency or strength-based studies have been conducted with Inuit in Canada overall?; and 3) what gaps in the literature exist for future research development? The findings will also be of relevance to Inuit scholars, Indigenous social service providers, and policy makers to inform efforts that promote health equity and improve health outcomes of Inuit who inhabit the territory that is now known as Canada.

## Method

This study is undertaken using the scoping framework defined by the Canadian Institutes of Health Research (CIHR) [38] and outlined by Arksey and O’Malley [39] and Levac Colquhoun and O’Brien [40]. Consequently, our process to explore literature and analyse the results occurs over six stages: 1) identifying the research question; 2) identifying relevant studies; 3) conducting a study selection; 4) charting the data; 5) collating, summarising, and reporting the results; and 6) conducting a consultation exercise [39].

A graduate student conducted a comprehensive manual search of 15 databases, repositories, and journals (*Études Inuit Studies, Arctic Journal, Circumpolar Health Bibliographic Database, SAGE, Web of Science, Social Science Abstracts, PubMed, CINAHL, Google Scholar, JSTOR, MEDLINE (Ovid), Scopus, the International Journal of Indigenous Health, WinnSpace*, and *MSpace*), using a combination of search terms related to health and wellness, geographical location, and language groups (see Appendix A: Table A1). Medical terms were added based on the MeSH (Medical Subject Headings) guidelines to provide a more accurate and comprehensive list (see Appendix B: Table B1). The databases and journals were chosen to include multidisciplinary perspectives and to achieve the widest possible coverage of the literature related to Inuit health in our identified region. We extracted information related to the location of each study, the date of publication, the area of study, and the type of study (statistical or experiential), and then provided a summary of research (i.e., based on the purpose and recommendations of the paper) to understand the landscape of the literature within a customised spreadsheet for MIA.

**Table B1.** MeSH terms.

Chronic Disease	Mental Health	Nutritional Sciences	Maternal Health	Environment and Public Health	Social Determinants of Health
Chronic Illness Chronic Illnesses Illness, Chronic Illnesses, Chronic Chronically Ill Multiple Chronic Conditions Noncommunicable Diseases	Psychological Emotional Social Well-being Mental disorders Behavioural Mental illness Psychiatry and Psychology Psychological Phenomena	Body Mass Index (BMI) Metabolism Diet Genetic Metabolic Over-weight Dietetics	Maternal Welfare Women’s health Child health Child Welfare Children’s Health Services	Environmental Impacts Ecological and Environmental Phenomena Climate Abnormalities Public Health	Population Characteristics Health status Epidemiology Trends

Articles pre-dating 1996 were excluded since those articles pre-date the Royal Commission on Aboriginal Peoples that called for “initiatives to address social, education, health and housing needs, including the training of 10,000 health professionals over a ten-year period” [41] and prompted a shift in academic studies and priorities. A more recent document, *Building on Values: The Future of Health Care in Canada*, recommends “new initiatives to improve timely access to care … and a special focus on the health needs of Aboriginal peoples” [42], p.xvii). This document also encourages Indigenous people to be involved in the process of reimagining healthcare, an important component of self-determination for MIA, which initiated this review.

Our initial search returned 143 articles. Studies were first screened by the date of publication. All studies that pre-dated 1996 were removed from the results. Articles were then screened by title, abstract, and summary of the research based on inclusion and exclusion criteria (see Appendix A: Table A1). Studies, which comprise articles in peer-reviewed journals, grey literature, and dissertations and/or theses that were populated from the search terms, were all included in the initial results. Studies were then screened by location in the title and abstract to include only studies that occurred within our geographical parameters. Once a study was confirmed to meet the two basic screening parameters, a result was populated in a spreadsheet. The most common reasons for exclusion during title and abstract review were: 1) the study was conducted in Nunatsiavut (Northern Labrador), and 2) the study pre-dated 1996. Finally, studies were reviewed in depth by two authors to confirm that a minimum of one paragraph was written about Inuit health and wellness within studies conducted in relation to Inuit located in Manitoba or the Kivalliq region after 1996. There were no conflicts between the two authors on the basis of criteria. The final results include 104 articles, spanning from 1996 until the data was collected in April 2019

## Results

Following the data collection and screening, six categories were identified and each article was coded according to these categories: Chronic Conditions and Diseases; Population and Preventative Health; Maternal Health; the Determinants of Health as proposed by the Public Health Agency of Canada (PHAC) (See Appendix C: Table C1), which was corrected to the Determinants of Inuit Health; Environmental Health; and Food or Nutrition-Based Health. Consistent themes that emerged throughout the study overwhelmingly focused on “negative and crisis-oriented experiences” with only six being identified as resiliency or strength-based studies. To code a study strength-based, the research project must be initiated by Inuit, the data collected must be interpreted by Inuit, and/or the study must use empowerment-based approaches within the methods section of the literature. Most articles from this review were published in an academic setting by authors who were affiliated with a university. Articles focused on Inuit health have been increasing; five articles, all published in 2014, were captured in our review compared to nineteen articles published in 2018 and seven articles published by April 2019. Overwhelmingly, the articles focused on the broad areas of nutrition, maternal health, and chronic illness, with a specific focus on mental health and death by suicide.

### Chronic disease

Chronic health conditions or diseases affect 44% of Canadian adults over the age of twenty, which are often managed through medical interventions [43]. Examples of chronic disease studies include tuberculosis, cardiovascular diseases, and mental illness [44]. Nineteen of 104 studies were coded Chronic Disease (18.3%), and the majority of these were reporting on suicide (and/or mental health) and tuberculosis.

#### Suicide

There were seven articles identified in the literature that focused on the prevalence of suicide in the included geographical location. These results are likely impacted by the fact that Inuit in Canada have the highest death by suicide rates in the world [22] and that “the average life expectancy in Inuit Nunangat is 10 years less than the Canadian average, due in part to suicide” (145) p.18). There is also a higher rate of hospitalisation due to self-injury [45 p.9]. Data collected from the Qikiqtani General Hospital reveals “that injuries caused by suicide attempts account for almost half of all the injuries among people age 20–29” [46 p.12] and “more than 4,000 incidents of assaults and intentional self-harm were also reported” [47 p.3].

The Truth and Reconciliation Commission of Canada’s 94 Calls to Action have detailed in Call Number 19 the need for Canada to work with Indigenous Canadians to close the gaps in health outcomes between Indigenous and non-Indigenous communities, using suicide as one of the indicators [48]. The often isolated geographical location of the Inuit population is intertwined with health delivery and outcomes. As O’Neill and colleagues state, “Nowhere is the formal and informal delivery of mental health support more complex in Canada than in the northern areas” [49 p.124]. The challenges and barriers in the northern communities include “professional isolation, managing confidentiality and negotiating multiple relationships – a situation involving a complex tangle of over-lapping personal, professional relationships, and community expectations” [49 p.125].

The themes of youth suicide and youth suicide prevention were prevalent in the research studies and grey literature captured in the results. Disclaimers appeared in some articles, noting that the national statistics for suicide are presented based on data of “status” or “registered” Indigenous people and Inuit residing in the Northwest Territories, leaving out non-status First Nations, Métis, and Inuit people living elsewhere [50]. The Nunavut Suicide Prevention Strategy began community consultations and conducted extensive research in 2008 [51]. In October 2010, the Nunavut Suicide Prevention Strategy was tabled in the Legislative Assembly of Nunavut, and by September 2011 the *Nunavut Suicide Prevention Strategy Action Plan* document was released, providing a full set of measures aimed at suicide prevention, intervention, and “postvention (i.e., support following attempted suicide or death by suicide)” [52], p.1). Overall, the evaluation report of the *Nunavut Suicide Prevention Strategy Action Plan* document found progress was being made towards the fulfilment of all eight commitment areas of focus^2^ ^2^These areas are: focused and active approach to suicide prevention; strengthened continuum of mental health services; youth skills; suicide prevention training; research on suicide and suicide prevention; communication and information sharing; healthy development in early childhood; and community development activities. and the objectives associated with these areas; however, the report concluded by stating “the overall vision for the Strategy is not being achieved at this time. There is no evidence that rates of suicide in Nunavut are decreasing” [52 p.8], and offering 42 recommendations for improvement.

Following the *National Aboriginal Youth Suicide Prevention Strategy (NAYSPS) Program Framework* that was released in 2013 [53], the Nunavut Chief Coroner held a Discretionary Inquest into suicide in Nunavut in September 2015. This documented highlighted that “Inuit communities know what is best for their youth and have identified the importance of community-based approaches to suicide prevention programs and other mental wellness activities in Inuit regions” [53 p.4]. Acknowledging community-based approaches to suicide prevention was echoed in other articles [54,55]. Despite the popularised idea of “elderly Inuit voluntarily abandoning their lives to the elements so as not to burden their surviving relatives”, the greatest risk of death by suicide occurred in males aged 15–29 years of age [55 p.1].

In July 2016, Inuit Tapiriit Kanatami (ITK), a non-profit organisation in Canada that represents over 60,000 Inuit, released the *National Inuit Suicide Prevention Strategy* (NISPS) [51]. Despite the fact that “there is no statistical information about the rate of suicide among urban Inuit” [56 p.10], the *National Inuit Suicide Prevention Strategy* underscores that the high rates of suicide “are a symptom of the social and economic inequities that have existed between Inuit Nunangat and most other regions of Canada since Inuit began to be impacted by colonization and transition of the land into permanent settlements” [56 p.5]. The immense impacts of residential and day schools on Inuit families and forced relocation provide context for the disparities in statistical indicators of health and wellness. The *NISPS* also detailed how the social determinants of health affect social equity, which if addressed, can be a suicide prevention strategy.

The *Qaujivallianiq Inuusirijauvalauqtunik – Learning from Lives that Have Been Lived: Nunavut Suicide Follow-Back Study* retrospectively examined the lives of all 120 Inuit who died by suicide in Nunavut between 2003 and 2006 and a comparison group of individuals with the same demographic background. The aim was to “identify risk and protective factors associated with suicide” [46 p.17]. The report concluded that “unemployment, childhood maltreatment, sexual abuse, impulsiveness, aggression, current and lifetime diagnoses of major depressive disorder, alcohol abuse or dependence and current or past cannabis abuse or dependence are risk factors for Inuit suicide in Nunavut. As such, there is an urgent need to provide better quality mental health care, counselling and substance abuse services for Inuit in Nunavut” [46 p.51].

#### Tuberculosis

There were two articles focused on tuberculosis captured in this review. Tuberculosis is an infectious airborne bacterial disease “caused by the organism Mycobacterium” (also known as Mycobacterium tuberculosis), which primarily attacks the lungs [57]. Statistics show “the average annual rate of TB among Inuit in Canada is more than 290 times higher than Canadian born non-Indigenous people” [58]. It is important to recognise the statistical data showing the high rates of TB among Inuit are rooted in the social determinants of health, including an inadequate physical environment and inequitable access to health care. Notably, 26.2% of Inuit live in a dwelling that is in need of major repairs, and this rate increases to 31.5% for Inuit living in Inuit Nunangat [1].

Tuberculosis has had devastating consequences for Inuit for generations, and in the 1950 s “at least one-third of the Inuit population was infected with TB” [59 p.xxi]. Inuit were removed from the community for years at a time to seek treatment in southern institutions often without consent [60]. Not only did this impact their familial and community ties, but it also affected their cultural roles and the experiential learning environment for children, which was necessary to prepare them with essential survival skills for the living conditions of Inuit Nunangat.

In October 2017, the Honourable Jane Philpott (then Minister of Health) and Natan Obed, President of Inuit Tapiriit Kanatami, announced the establishment of a Task Force to develop an *Inuit Tuberculosis Elimination Action Framework* [58]. TB rates correlate with many social determinants of health, “including inadequate housing, food insecurity, poverty, and stigma” [61]. The framework details six priorities, including the enhancement and strengthening of TB care with prevention programming; reduction of poverty; improvement of the social determinants of health; creation of social equity and empowerment within communities; development and implementation of Inuit specific solutions; and assurance for accountability in TB elimination.

### Population, public, and preventative health

Articles coded under population and preventative health included tobacco use, blood pressure studies, access to healthcare, and immunisations or vaccinations. There was a total of seven articles included in this category (6.7%).

Cancer is a leading cause of death among Inuit living in Canada [62]. Nunavut has the highest proportion of smokers over the age of twelve in all of Canada, and consequently, lung cancer is the most common cancer in the region [63]. Lung cancer can also be the effect of environmental exposure to smoke (more commonly known as second-hand smoke) or stone carving dust (which can contain the known carcinogens asbestos and silica). We did not capture any studies that focused on lung cancer rates and environmental exposure. The incidences of lung cancer are significantly higher in Inuit than in non-Inuit [63] and cervical cancer rates, are strikingly higher in the Kivalliq region than other parts of Nunavut [63]. Pauktuutit Inuit Women of Canada developed *Inuusinni Aqqusaaqtara – My Journey*, a cancer resource to increase Inuit knowledge about cancer and provide resources to patients, families, caregivers, and health care professionals. Tobacco use is also an important risk factor not only for cancer, but also tuberculosis, which “remains a significant public health problem in Canadian Inuit communities” with incidence rates of tuberculosis thirty-five times the Canadian average [64] p.1)

Although hypertension (high blood pressure) is a chronic disease, blood pressure more generally is not. Some studies have reported lower blood pressure among the Inuit [65–68]; however, one study from our results stated, “The highest blood pressures were found among the Kivalliq Inuit” [69] p.95), and directs future research to explore the impacts of traditional diet, a rural lifestyle with a low level of psychosocial stress, and genetics which may be factors influencing results. This study also analysed the effects of “factors like age, gender, obesity and smoking” on blood pressure [70 p.92] and concluded that these are risk factors for Inuit across the circumpolar region [70 p.98].

Geography impacts population health as well as recruitment and retention of trained staff within the remote medical facilities. Through exploring frontline staff experiences, McDonnell and colleagues documented “non-clinical determinants of medevac decision-making” [71 p.8]. Medevacs transport a patient by plane or helicopter to access health services usually in more urban settings. They found that “[r]ecruitment and retention of permanent [healthcare] staff” [71] p.9) would decrease the use of Medevacs to only critically necessary health emergencies.

There were two statistical studies reviewing the vaccination rates in Nunavik (Northern Quebec) [72; 73] which were located outside the regional scope of this review. These studies can provide direction for similar research to be conducted within Northern Manitoba or the Kivaliq region of Nunavut. In 2002, a mass immunisation campaign was launched to “control an outbreak caused by a virulent clone of serotype 1 Streptococcus pneumonia” [72 p.36]. Results determined this intervention was “short lived” in controlling invasive pneumococcal disease (IPD). Ultimately this study concluded that “effective pneumococcal vaccines and socioenvironmental strategies are needed to eliminate geographic disparities in IPD risk” [72 p.41]. In another study, Le Meur and colleagues focused on Otitis media (OM), a common ear infection caused when the space behind the eardrum fills with fluid [73]. OM is considered “an important public health problem in the Inuit population of Nunavik, Northern Quebec” [74 p.5180], and the study reviewed rates of OM and the variable use of pneumococcal conjugate vaccine (PCV).

Despite being outside the regional scope of this review, one study used samples from the 2004 Nunavik Inuit Health Survey, and explored the possibility of rabies virus-neutralising antibodies (rVNA) in Inuit but ultimately concluded results “found no evidence of rVNA among Inuit adults in Nunavik” [75 p.6].

### Reproductive and sexual health

#### Sexual health

Our scoping review reveals that there are few studies on sexual health among Inuit. In 2017, Pauktuutit, the national non-profit organisation representing all Inuit women in Canada, released a report titled *Tavva: National Inuit Sexual Health Strategy*. The report advocated for Inuit involvement in design, delivery, and evaluation of culturally and linguistically appropriate campaigns to create healthy Inuit sexuality programs [76]. This report discussed the chlamydia rate in Nunavut, which is higher than the national average. The gonorrhoea rate in Nunavut is over fifty times the national average [76]. Although both of these statistics are not Inuit-specific, the population of Nunavut is approximately 85% Inuit. The lack of Inuit-specific studies focused on sexually transmitted disease and illness is a major gap in the literature.

#### Maternal and child health

Twenty-nine studies captured in our review focused on maternal health (27.9%); however, only two studies used primarily qualitative methods and focused on Inuit women’s experience as patients during care [77; 78]. In one study, interviews were conducted with both First Nations and Inuit women who were identified as having medically “high-risk” pregnancies that resulted in their transfer to southern hospitals for maternal care [77]. Although this study interviewed women from Northern Quebec, medical evacuation is standard practice for Inuit (and First Nations) women living in rural and remote regions throughout Canada and is said to reduce the high rates of maternal and infant mortality among Indigenous women [78; 79] and improve perinatal outcomes [80].

The second qualitative study centred on the theme of teenage pregnancy [81]. The study revealed the common practice of “customary adoption” where the parents of the pregnant mother or another family member will adopt the baby. Another area of focus within this report was abortions, especially among young mothers. Using data from the Nunavut Statistics, which are, in turn, based on Statistics Canada data, it should be noted that the rates presented on the prevalence of abortions performed were for all women in the geographical location and not limited to Inuit women.

One study examined the notion of “lost births,” a term used to explain “the loss of … community knowledge and celebration regarding the birth of a new member” [82 p.70]. The study goes on to argue that “lost births are a result of historical, systemic oppression of Indigenous culture by the Canadian Government, and the monopoly of formal health education and services by the medical profession” [82 p.70]. The paper concludes by recommending support, including sustainable funding, for Indigenous midwifery practice.

Another research study, which was largely concentrated in the Kivalliq region of Nunavut from November 2002 to December 2004, highlighted the need to return birth home to provide sustainable maternal and perinatal care. Communities are seeking ownership over the design, development, and evaluation of care: “This is not simply about community-involvement [in maternity care] but community ownership and participation in a partnership with other stakeholders as equals” [83 p.14]. A midwifery model of integrating traditional Inuit knowledge and modern medicine is offered in the maternity department at Centre de santé Inuulitisivik in Puvirnituq since its creation in 1986. Focused on seven communities north of the 55th parallel (Kuujjuarapik, Umiujaq, Inukjuak, Puvirnituq, Akulivik, Ivujivik, and Salluit), the work of midwives has allowed approximately 92.2% of deliveries to occur in Nunavik, while only 7.8% of women had to travel to Montreal to give birth [84]. The Centre observes that “[t]he presence of midwives in the north has allowed Inuit communities to reclaim the experience of pregnancy and childbirth, avoiding the separation of families” [84]. In a research study from 2010, results confirmed that “in Nunavik … risks of perinatal death were somewhat but not significantly higher in the Hudson Bay communities where Inuit midwives served as the primary birthing attendants compared with the Ungava Bay communities where western physicians served as the primary birthing attendants” [85].

Turning from the health and wellness of pregnant women to that of newborn infants, in Nunavut, the rate of infant mortality is consistently higher than for the rest of Canada [86]. Using statistical data gathered from 2002–2012 found “the proportion of live births between 28 to 36 weeks is an average of 1.7 times higher in Nunavut than in the rest of Canada” [86 p.4].

In late summer and fall of 2007 and 2008 research was collected in sixteen communities within Nunavut, which informed *The Nunavut Child Inuit Health Survey* of children, ages 3–5. This research focused on five themes to identify the most pressing issues faced by the communities: home environment, food insecurity, general health; nutrition and food habits; and respiratory illnesses [87]. Many of these themes were found throughout the studies captured in this review.

#### Fetal alcohol spectrum disorder (FASD)

Inuit have identified community-led early childhood development and prenatal care as priorities for decades, encompassing awareness campaigns for Fetal Alcohol Spectrum Disorder (FASD). The 2006 Aboriginal Peoples Survey reports that ﬁve per cent of those Inuit living outside of Inuit Nunangat have been diagnosed with FASD [88 p.3]. This theme appeared most often in grey literature. In 1996, Pauktuutit Inuit Women of Canada conducted “an information-sharing and skill-building workshop on FASD,” which resulted in a final report [88 p.4]. The same year, Pauktuutit prepared a resource guide, *Foetal Alcohol Syndrome: A Resource for Inuit Communities to Understand What FAS is and What They Can Do to Help*. In 1997, Health Canada released *It Takes a Community: Framework for the First Nations and Inuit Foetal Alcohol Syndrome* and *Foetal Alcohol Effects Initiative, A Resource Manual for Community-based Prevention of Foetal Alcohol Syndrome and Foetal Alcohol Effects* [89]. Yet another Fetal Alcohol Spectrum Disorder (FASD) resource guide was prepared in 1998 entitled *Ikajuqtigiinniq*, which was based on a national Inuit FASD workshop.

Pauktuutit have been the leader in knowledge mobilisation related to FASD. In 2001, Pauktuutit released a media campaign, which included posters, radio spots, and a video targeted at Inuit youth to highlight the consequences of consuming alcohol during pregnancy. An Inuit-speciﬁc FASD health promotion resource kit called *Children Come First: A Resource About FASD* was disseminated in 2003. In 2010 Pauktuutit released the *Inuit Five-Year Strategic Plan for Fetal Alcohol Spectrum Disorder 2010–2015 Report* [88]. This report aims to “enhance FASD prevention and diagnosis and to support the needs of individuals and families living with FASD in Inuit communities” [88 p.6]. The report identifies “a range of permanent, holistic, responsive support services for families and FASD-affected Inuit” [88 p.26] that are offered in layers to the community.

#### Healthy diet for mothers and infants

Our review captured a total of 21 articles related to a healthy diet for Inuit mothers and infants. Following the application of exclusion criteria, eight studies were removed based on their geographical focus.

In response to the 2006 Aboriginal Children’s Survey (ACS), which reported breastfeeding initiation for all Inuit children was less compared to the rest of Canada, Asuri and colleagues, including Inuit Tapiriit Kanatami representatives, produced their own report [90]. Taking into account customary adoption, Inuit children in the care of the biological mother have breastfeeding initiation rates of 76% [90]. The remaining studies focused on statistics or mixed methods approaches to research. One study examined the prevalence of vitamin D in lactating Inuit women living in the Arctic, who were participating in the 2007–2008 International Polar Year Inuit Health Survey [91]. Since traditional diets are high in vitamin D due to a “diet rich in fatty ﬁsh and marine mammals” [91 p.1988], this study sought to measure the current levels of vitamin D in lactating women. Results reveal “healthy maternal vitamin D status was observed in 16% of participants during the late summer and early autumn” [91 p.1993].

Another vitamin-related maternal study was conducted in relation to red blood cell folate (RBCF) [92]. Folate is a type of B vitamin found in food as the natural form, whereas folic acid is the “man-made” version of this vitamin. This vitamin is required for growth, especially in a developing foetus. It is well documented that folate has an important role in preventing some birth defects, such as defects of the brain and spinal cord (also called neural tube defects [NTDs]) [93; 94; 12]. Results from this study suggest that folate status is often too low in Inuit women of childbearing years and “women would benefit most from supplemental folic acid fortification and other programs to improve folate status” [92 p.690].

#### Abuse

Maternal and child health is not limited to the development of the child. One study conducted in Nunavik found that one-third (35.8%) of Inuit experienced domestic violence (verbal or physical abuse) by the mother or her spouse at least once per month in the year following the birth of a child [95 p.67]. All “psychosocial difficulties such as … domestic abuse impacts a mothers’ understanding of their child’s needs, emotions, and intents” [95 p.65]. The prevalence of self-reported child sexual abuse was identified in the Community and Personal Wellness module of the *Inuit Health Survey 2007–2008*. Results showed that of the “1,710 Inuit respondents, 41% (52% of female and 22% of male respondents) said they had experienced severe sexual abuse during childhood, which includes someone threatening to have sex with them, touching the sex parts of their body, trying to have sex with them, or sexually attacking them” [56 p.21]. These findings could be linked to youth mental health conditions or to the risk of death by suicide rate as detailed above [22; 56,45,46; 50; 53; 54]. Additionally, “about one in four (27%) Inuit women in Nunavut reported having experienced some form of forced sexual activity [sexual assault] as an adult [96]” (p.10).

### Social determinants of health

The social determinants of health are predominant in the literature with fifteen studies coded under that theme (14.4%). In 2014 Inuit Tapiriit Kanatami (ITK), with input from their National Inuit Committee on Health (NICoH) and in partnership with the Inuit Public Health Task Group, released *The Social Determinants of Inuit Health in Canada* report [17]. This report identified eleven factors as key social determinants of health, including quality of early childhood development; housing; culture and language; livelihoods; income distribution; personal safety and security; education; food security; availability of health services; mental wellness; and the environment. Of these factors, most of the research found in our scoping study addresses the environment and food security.

According to the World Health Organisation, “the social determinants of health (SDH) [sometimes referred to as SDOH], are the conditions in which people are born, grow, work, live, and age, and the wider set of forces and systems shaping the conditions of daily life. These forces and systems include economic policies and systems, development agendas, social norms, social policies and political systems” [18]. Upon European arrival, Inuit transitioned from nomadic lifestyles “to working and living in communities year-round” [97 p.201].

#### Housing

Studies have found that Inuit living in Inuit Nunangat are more likely to live in a crowded dwelling compared to the wider Canadian population [13]. According to Patterson and colleagues, “half (52%) of all Nunavummiut [Inuit] live in social housing and, depending on the community, up to 72% of those are living in overcrowded housing. Housing can be so crowded that some residents sleep in shifts, as it is all too common for more than 20 people to call a 4-bedroom house a home” [58 p.83].

#### Culture and language

Language is identified as a strong determinant of Inuit health [17]. In 2008, the Inuit Language Protection Act was adopted by the Government on Nunavut [98] affirming the “inherent right to the use of the Inuit Language, and that positive action is necessary to protect and promote the Inuit Language and Inuit cultural expression, and is consistent with Canada’s international undertakings, including the International Covenant on Civil and Political Rights, the International Covenant on Economic, Social and Cultural Rights and the Convention on the Protection and Promotion of the Diversity of Cultural Expressions, proclaimed by the United Nation” [99]. Inuktitut remains one of the most prominently spoken Indigenous languages, and according to one report, in Kivalliq and Baffin, 80% of children spoke Inuktitut [87]. This is a positive display of resiliency by maintaining culture that is embedded within language [17]; however, language barriers do occur at nursing stations “since few nurses in the north are Inuit” [100].

#### Environmental health

Related to language and culture, traditional knowledge (TK) is “based on intergenerational transmission of knowledge and oral history on human – environmental relationships, personal and community well-being, and spiritual considerations, remains a key feature of Inuit life” [101] p.e13). The Inuit way of life has been impacted negatively by the effects of climate change, drawing international attention [102]. Eight articles (7.7%) that study environmental health in relation to Inuit include the impacts of climate change on population health [102]; climate change as it relates to mental health (103; 104); evaluation of public funding for health projects related to climate change [105]; and water contamination which was linked to acute gastrointestinal illness [106]. As noted in one Truth and Reconciliation Commission document, environmental health and food security issues are connected to the broader context of the child-welfare crisis [23 p.191].

Environmental changes from human interference, including pollutants, can have serious health impacts. Persistent Organic Pollutants (POPs) are “a group of chemicals that are toxic and can cause cancer and other adverse health effects [to animals and humans]” and “travel vast distances via air and water” [107]. Trends in the arctic air were evaluated and provided evidence that “national and international control measures applied both before and since the establishment of the Stockholm Convention (SC) of contaminants” were successful [108]. The Stockholm Convention is “a global treaty to protect human health and the environment from [38] highly dangerous, long-lasting chemicals by restricting and ultimately eliminating their production, use, trade, release and storage” [109 p.1]. POPs therefore do not only influence human health, but with their effect on animals can impact local food sources.

Using hair analysis and questionnaires, contaminants such as methylmercury (MeHg) were documented in 1,145 individuals from three Inuit communities in Labrador [110]. MeHg exposure has also been associated with cardiovascular health risks for adults [111–114]. Evidence for cardiovascular disease in the Inuit population is relatively unexplored with inconsistent results [115]. The environmental conditions and health are intertwined and indivisible.

#### Food security and nutrition

A predominant theme throughout the research was nutrition and food insecurity with 26 studies (25%) included in this review. Food security is defined as having “physical and economic access to sufficient, safe, and nutritious food to meet … dietary needs and food preferences for an active and healthy life” [116] p.1). Due to the geographical location (another social determinant of health), food access and cost impact the overall diet of many Inuit living in the north. In some coastal northern communities, the shoreline waters are only open for as little as two months per year due to the extremely cold temperatures [59]. Food import via water access is therefore limited and other modes of transportation may be costly. As the Truth and Reconciliation Commission states, “In many Inuit communities, healthy food is very expensive” [117 p.43]. Food prices in Nunavut are, on average, “twice those in southern Canada” [58 p.6]. Food is also limited in terms of choice, and occasionally further limited by the inevitable difficulties of transporting food long distances by air [58].

According to the Truth and Reconciliation Commission, at the peak of residential schools, “children who were used to eating raw and frozen meat had to make an even more difficult transition” [118 p.27] to a different diet due to food scarcity. Food security for Inuit has been well documented in the literature [13; 119; 120]. Just over one third (35%) of Inuit aged 25 to 54 years who had low or very low food security were predicted to be in excellent or very good health, after accounting for other factors according to the Aboriginal Peoples Survey [121]. Diet can be linked to income, which is another social determinant of health.

Some food-related studies were linked to maternal and child health [122,123,124,125,126,127,128; 92]. The psychometric evaluation (a standard scientific method used to measure individuals’ mental capabilities and behavioural style) of a modified Household Food Insecurity Access Scale (HFIAS) was explored [122]. The HFIAS was designed to assess food insecurity among pregnant Inuit women from Nunavik (an area of Northern Quebec). The modified HFIAS instrument was considered useful to measure food insecurity among pregnant Inuit women from Nunavik and “inform interventions targeting health conditions impacting groups that obtain food through both monetary and non-monetary means” such as hunting and fishing [122 p.2].

A few of the studies focus on the impact of nutrition on chemical levels of pregnant Inuit women. One study investigated the levels of organochlorines (OCs) in infants from their mothers’ diets and consumption of fish and marine mammal fat [128]. Another study examined “the relationships between socio-economic characteristics and mercury (Hg) and polychlorinated biphenyl (PCB) concentrations among pregnant Inuit women from Nunavik.” [129].

A community-based, multi-institutional nutritional and lifestyle intervention – Healthy Foods North – was implemented to improve dietary intake among Inuit and Inuvialuit in four intervention communities in Nunavut and the Northwest Territories in 2008 and aimed to reduce the risk of chronic disease in the North [130]. Results concluded that community-based and community-driven nutrition intervention programs have a greater potential to “reduce weight and related health risks than individual weight loss programs” [131 p.8] and the Healthy Foods North program was “effective for reducing consumption of high fat, high sugar foods and beverages of low nutritional density and reliance on preparation methods that add fat to foods” (p.9).

## Discussion

The purpose of this scoping review is to summarise previous Inuit health and wellness studies in Manitoba and the Kivalliq region of Nunavut in order to provide a snapshot of the types of studies available and identify the gaps in knowledge. This review is done in collaboration with the Manitoba Inuit Association (MIA) in a partnership with The University of Winnipeg through the CIHR NEIHR program. As a research partner, MIA is working towards building their own research policies for future partnerships and this research supports their work as they *themselves* identify areas of research that would most benefit their communities. This self-determination in research by Indigenous partners is the basis of the larger NEIHR project and supports the *National Inuit Strategy on Research* that argues, “Inuit have the greatest insight on the nature of the challenges facing Inuit families and communities, coherent ideas for how to address those challenges, and the strongest incentives to use research as a tool for developing and implementing innovative solutions to society’s problems” [132 p.11]. Research to date has largely been disease-based and has often provided comparisons between Indigenous and non-Indigenous people. Distinct Inuit experiences are rarely written about from an Inuit perspective where the authors have chosen to self-identify as Inuit. Despite this gap, Inuit Tapiriit Kanatami and Pauktuutit Inuit Women of Canada have been leaders in strengths-based community research and publications that address priorities as determined by Inuit.

There were some obvious areas within preventative health that may benefit from further investigation. Despite a significant collection of literature focused on suicide, gaps appear in the rates of suicide attempts and suicidal ideation (thoughts of committing suicide), research that could lead to new prevention strategies or ways of understanding mental health. Sexual health was mostly absent in the literature, and future research topics could include an evaluation of harm reduction strategies or trauma-informed approaches, including culturally appropriate education for the prevention of sexually transmitted diseases or infections. Future exploration of stigma and shame in sexual health would also be beneficial.

Additionally, research on maternal and child health was limited. Although there were studies and an advocacy report for “bringing birth home,” evaluation of Inuit midwifery and traditional birth attendants could be further explored. The role of fathers has also been excluded from the current literature, and more information on breastfeeding is necessary.

Under the *Social Determinants of Inuit Health* framework, future research could address familial connections since the involvement of family is a central feature of Inuit culture [17]. Oral health is also an area that has been identified as an ongoing issue in Inuit Nunangat [133]; however, given the efforts of CIHR in improving Indigenous oral health, this area may eventually have an expanded focus in the literature.

Although this paper and many studies captured within its parameters tend to focus on negative experiences of living in remote, isolated, and environmentally harsh conditions, it is imperative to recognise Inuit’s extreme resiliency needed to withstand and adapt to these conditions. Colonialism has created and sustained social inequality for Inuit, and government and government-sponsored systems and structures, such as “Indian hospitals,” impacted Inuit (among other Indigenous populations) with untreated heath conditions and exposure to highly contagious infections [134]. These structures perpetuated racial inequalities, and ultimately, created health disparities that continue today. As research in Indigenous communities continues to focus on self-determination in research and to support strength-based inquiry, acknowledging structural inequity as the basis for diminished health is essential.

## Appendix A

**Table A1.** Screening study parameters.

Health and Wellness	Indigenous	Region	Inclusion Criteria	Exclusion Criteria
Maternal health Child/Youth health Chronic Diseases/Conditions Mental health Addiction Nutrition/Diet Health promotion Community-based Traditional Medicine Alternative approaches to Health and Wellness	Inuit Innu Inuk* Nunavummiut Eskimo Arctic Indigenous	Manitoba Nunavut Kivalliq region Keewatin Inuit Nunangat Inuvialuit Circumpolar Arctic	Location was within the identified region Indigenous identity matched the criteria Minimum one paragraph on Inuit Health and Wellness	Regional or national studies that do not specifically mention Inuit Identified as Yupik, Kalaallit Aleut, or Inupiat people Studies conducted in Greenland, Russia, Alaska, or other areas outside of the scope

## Appendix B

**Table C1.** Determinants of Health proposed by Public Health Agency of Canada (PHAC).

Determinants of health are a comprehensive range of personal, social, economic and environmental factors that determine and influence health.
Income and social status Employment and working conditions Education and literacy Childhood experiences Physical environments Social supports and coping skills Healthy behaviours Access to health services Biology and genetic endowment Gender Culture
